# Recent Developments in Catalytic Carbonyl Sulfur Hydrolysis

**DOI:** 10.3390/ma18051097

**Published:** 2025-02-28

**Authors:** Zongshe Liu, Yinjuan Dong, Chenghua Xu, Feng Chen, Wenzhu Liu, Yan Yang, Lingyu Guo

**Affiliations:** 1 Research Institute of Natural Gas Technology, PetroChina Southwest Oil & Gasfield Company, Chengdu 610200, China; liu_zsh@petrochina.com.cn (Z.L.); chenfengcf1994@petrochina.com.cn (F.C.); liuweizhu@petrochina.com.cn (W.L.); 2 College of Resources and Environment, Chengdu University of Information Technology, Chengdu 610225, China; xch@cuit.edu.cn (C.X.); yangy@cuit.edu.cn (Y.Y.); gly15196454714@163.com (L.G.); 3 Air Environmental Modeling and Pollution Controlling Key Laboratory of Sichuan Higher Education Institutes, Chengdu 610225, China

**Keywords:** carbonyl sulfide, catalytic hydrolysis, catalyst, evaluation metrics, reaction mechanism

## Abstract

Carbonyl sulfide (COS) is the most abundant and longest-lasting organic reduced sulfur compound in the atmosphere. Removing it is a critical and challenging aspect in desulfurization technology in order to comply with global restrictions on harmful emissions. Catalytic hydrolysis refers to the process whereby COS reacts with water under the influence of a catalyst to generate carbon dioxide and hydrogen sulfide. Due to its high conversion rate, minimal side reactions, no hydrogen consumption, and mature technology, it has emerged as the most crucial COS removal method at present. Since its inception in the 1940s, research on the catalytic hydrolysis of COS has witnessed encouraging progress over the past several decades. This review summarizes recent advancements in this field. In this review, the evaluation metrics, influencing factors, and reaction mechanism for the COS hydrolysis reaction are briefly introduced. The recent advancements in COS hydrolysis catalysts in recent years are emphasized. Additionally, the existing challenges and potential solutions in this field are also proposed. Finally, the future development directions for this research area are envisioned. The purpose of this review is to offer a reference for the subsequent design and research of high-activity and high-stability hydrolysis catalysts.

## 1. Introduction

With the rapid advancement of the economy, the contradiction between energy supply and demand has grown increasingly prominent, accompanied by a substantial increase in the exploitation of natural resources, which has given rise to numerous environmental issues. Consequently, the scientific utilization of high-sulfur-containing energy sources has attracted considerable attention [1,2,3]. Typically, chemical feedstock gases are derived from natural gas, petroleum, and coal, and the sulfides produced during their preparation can generally be classified into two categories: organic and inorganic sulfur. Hydrogen sulfide (H_2_S) is the primary form of inorganic sulfur, while organic sulfur mainly comprises carbonyl sulfide (COS), mercaptans, thiophenes, and carbon disulfide (CS_2_). Among these, COS, as a toxic and harmful gas [4], accounts for approximately 80–90% of the total organic sulfur and boasts the longest atmospheric lifetime of about two years, making it the most persistent reduced sulfur compound in the atmosphere [4,5,6]. Given COS’s chemical inertness, it presents new challenges to existing desulfurization technology [7].

During the chemical production process, the existence of COS not only affects the quality of chemical products, but also corrodes gas pipelines, shortens the service life of equipment, causes catalyst poisoning, and increases production costs [8,9,10]. The adsorption of only 4 mg of sulfur per gram on the surface of the Fe-CuK catalyst reduces the activity of the Fischer–Tropsch process by approximately 50% [6]. Additionally, untreated COS emissions into the atmosphere are prone to oxidation into sulfur dioxide, which leads to environmental problems such as acid rain and photochemical smog, posing significant risks to human health. Notably, the aforementioned problems can be thoroughly resolved via the catalytic hydrolysis route, which can be achieved in highly efficient, durable, and gentle ways in the presence of appropriate catalysts [10,11].

COS demonstrates lower solubility in water compared to carbon dioxide, but dissolves rapidly in alcohol, alkaline alcohol solutions, and toluene. This colorless, odorless, and toxic gas is omnipresent in industrial gases such as water gas, liquefied petroleum gas, natural gas, coke oven gas, and tail gas [12]. During sulfur-containing gas treatment processes, the properties of COS are stable. Therefore, it is challenging to effectively convert or remove COS through conventional desulfurization methods. Currently, the main methods for removing COS include reduction, absorption, oxidation, photolysis, and hydrolysis [6,13,14,15,16]. For instance, aqueous alkanolamine solutions can be utilized for absorption, but this method is relatively complex and has a low reaction rate. Some alkanolamine solutions cannot be regenerated after adsorption [17]. It has also been proposed that these solutions be employed to synthesize photocatalysts, a novel approach that is still far away from being used in potential industrial applications [18]. The principle is that COS reacts with water to generate carbon dioxide and hydrogen sulfide under the action of a catalyst. In 2002, Svoronos and Bruno provided the reaction formulation (COS(g) + H_2_O(g) → CO_2_(g) + H_2_S(g)) [19]. Compared to other methods, COS hydrolysis has the advantages of a low reaction temperature, zero consumption of hydrogen, and minimal side effects, and is thus is considered the most promising candidate among these potential methods [19,20].

**Figure 1:**
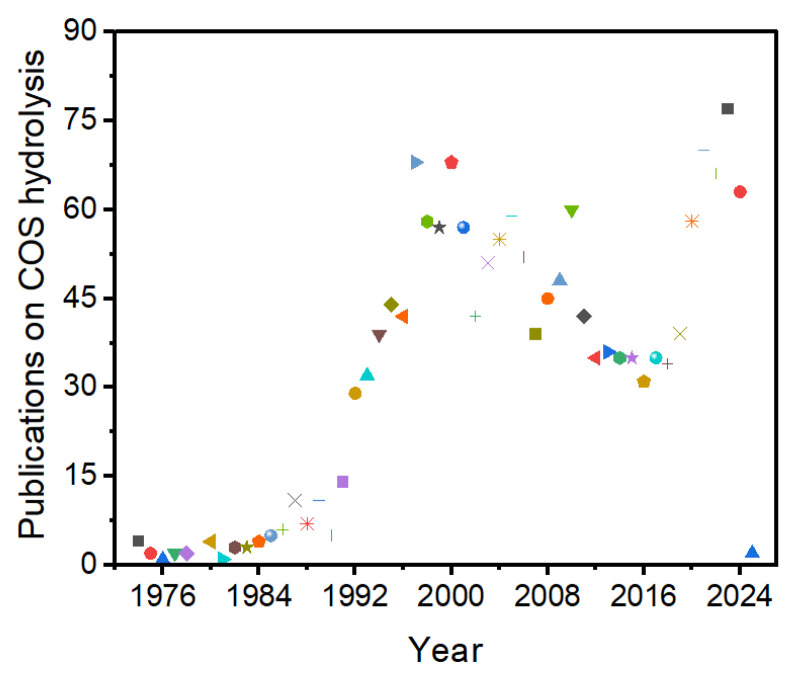
Increase in scientific interest toward COS hydrolysis.

COS hydrolysis originated in the 1940s and has gained momentum since then, as depicted in Figure 1. COS catalytic hydrolysis is regarded as an economical approach for converting non-removable COS into H_2_S, which can then be readily removed through a mature wet or dry desulfurization process [21]. As COS hydrolysis is an exothermic reaction, low temperatures are conducive to its thermodynamics. The rate and efficiency of COS hydrolysis are influenced by various factors, including the type and properties of the catalyst, the reaction temperature and pressure, and the composition of the raw gas. For instance, the choice of the active component and carrier of the catalyst significantly impacts the reaction performance. Increasing the reaction temperature typically accelerates the reaction rate but may also enhance side reactions. The reaction pressure can affect the adsorption of reactants and the desorption of products. COS hydrolysis technology is widely applied in complex atmospheres, such as CO, CO_2_, H_2_, O_2_, and H_2_S gases. Different compositions of the feedstock gas produce distinct atmospheric effects on COS hydrolysis, affecting the normal operation of the hydrolysis catalyst and process. Thus, studying and analyzing the atmospheric effects of COS hydrolysis are crucial for the application of new processes and catalysts.

Currently, global research on COS hydrolysis mainly focuses on two primary aspects: the development of hydrolysis catalysts and the exploration of reaction kinetics and mechanisms. Catalysts (such as metal oxides, supported catalysts, and hydrotalcite-derived materials) play a crucial role in COS hydrolysis. They can reduce the activation energy of the reaction and accelerate the reaction rate. Common COS hydrolysis catalysts comprise active components and supports. Active components generally include metal oxides [22,23,24], alkali metals [25,26], alkaline earth metals [23,27,28], transition metals [14,29,30,31], nano-metals [5,32,33], and rare earth metal oxides [7,34,35,36]. Carriers typically consist of activated carbon [37,38], titanium oxide [39,40], alumina oxide [34,41,42,43,44], etc. These catalysts are loaded onto the supports by means of impregnation, decomposition, etc., to form an efficient catalytic system. Meanwhile, the basic principle of COS hydrolysis involves multiple aspects such as the chemical reaction process, catalyst function, reaction mechanism, and influencing factors. The research on reaction kinetics and mechanisms can not only facilitate the development of high-performance and high-activity hydrolysis catalysts but also provide a theoretical basis for the design of reactors.

This study will systematically summarize the reaction devices, detection technology, evaluation metrics, influencing factors, and reaction mechanisms of COS hydrolysis. In particular, the research progress in relation to COS hydrolysis catalysts in recent years is emphasized. Furthermore, the existing challenges and potential solutions in this field are also presented. Finally, the future development directions of this field are explored. This review aims to provide in-depth perspectives and guidance to researchers in the broader scientific community interested in innovative green technologies for COS hydrolysis and catalyst design.

## 2. Evaluation Metrics of COS Hydrolysis Reaction

### 2.1. Evaluation of the Reactor System

**Figure 2:**
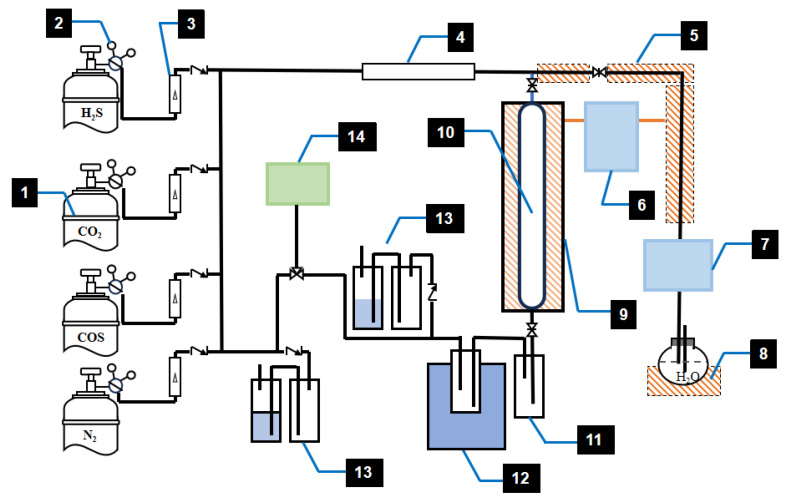
Layout of the experiment evaluating the activity of the catalyst (1—gas cylinder: H_2_S, CO_2_, COS, and N_2_; 2—pressure-reducing valve; 3—rotor/mass flowmeter; 4—gas mixer; 5—heating zone; 6—heating controller; 7—micro-injection pump; 8—water source; 9—fixed bed and heating furnace; 10—quartz reaction tube; 11—liquid product collection bottle; 12—cold trap; 13—exhaust gas treatment and anti-suction bottle; 14—online chromatography).

In this study, the water hydrolysis catalyst was employed at a medium temperature and atmospheric pressure. The experimental study on catalyst activity was conducted in a fixed-bed quartz glass reactor using a heating tube furnace. The experimental reaction temperature was controlled by an intelligent programmable temperature controller. During the experiment, the gas in each cylinder was regulated by rotameter/proton flowmeter and entered the buffer container for thorough mixing. Then, it was combined with the water output by a micro-dose pump and vaporized by the heating strip before entering the fixed-bed reactor containing the water-hydrolysis catalyst for reaction. The reactor was placed in a heating furnace controlled by an intelligent temperature controller. Subsequently, it entered a constant-temperature water bath for control and entered the water-saturated system, where water vapor was introduced into the reaction atmosphere. The saturated mixture entered the fixed-bed reactor and passed through the COS water hydrolysis catalyst. There were gas sampling ports at both ends of the reactor, and the exhaust gas outlet was at the bottom. The inlet and outlet concentrations of CO_2_, CO, and H_2_S were measured by a gas chromatograph specialized in sulfur analysis. The schematic diagram of the catalyst activity evaluation device is shown in Figure 2.

### 2.2. Evaluation Metrics of COS Hydrolysis

The COS reacts with H_2_O under the action of a catalyst to produce CO_2_ and H_2_S. The basic reaction equation is as follows (Equation (1)) [45].


(1) $$COS+H_{2}O\to CO_{2}+H_{2}S$$

The COS conversion and total H_2_S yields (THY) were obtained based on Equations (2) and (3).

(2) $$COS\;conversion\left(\%\right)=\frac{{\left[COS\right]}_{in}-{\left[COS\right]}_{out}}{{\left[COS\right]}_{in}}\times 100\%$$

(3) $$THY{\;H}_{2}S\left(\%\right)=\frac{{\left[H_{2}S\right]}_{out}}{{\left[COS\right]}_{in}}\times 100\%$$

where [COS]_in_ and [COS]_out_ are the concentration of COS in the inlet flue gas and outlet flue gas, respectively, and THY H_2_S represents the generation rate of H_2_S during COS hydrolysis.

## 3. Factors Affecting the COS Hydrolysis Reaction

There are a multitude of factors influencing COS hydrolysis, such as the catalyst, the reactant concentration and purity, the reaction temperature and pressure, the operating conditions, the design of the reactor, and the generation and inhibition of by-products [2,6]. In practical applications, it is of paramount importance to comprehensively consider these factors and enhance the efficiency and conversion rate of the COS hydrolysis reaction by optimizing the reaction conditions and process parameters.

(1) Catalyst

Different catalysts exhibit diverse catalytic effects on the COS hydrolysis reaction. Effective catalysts can lower the activation energy of the reaction, thereby accelerating the reaction rate. The selection of the catalyst, the preparation of reaction conditions, its activity and stability, and their regenerability are all critical factors influencing the hydrolysis efficiency. Additionally, the surface area, the pore size distribution, and the number and properties of active sites also have an impact on its catalytic performance. Catalysts may become deactivated over time due to contamination, sintering, or other reasons, thereby requiring regular maintenance and regeneration treatment to restore their catalytic activity. This aspect will be elaborated upon in the COS catalysts section.

(2) Reactant Concentrations

COS concentration: The reactant concentration of COS directly influences the reaction rate and conversion rate. Generally, the higher the concentration, the quicker the reaction rate. However, excessively high concentrations can lead to catalyst poisoning or the increased formation of byproducts.

Water content: As one of the reactants, the water content also affects the reaction outcome. An appropriate amount of water facilitates the reaction, while excessive water can dilute the reactant concentration, reducing the reaction rate. Li et al. [1] discovered that the competitive adsorption between COS and water at the active site leads to the negative impact of water on COS hydrolysis rates. By blocking access to the active site, the adsorbed water can function as an inhibitor of the COS hydrolysis reaction. As the relative humidity in the feed increases, the ability to form a water film on activated carbon significantly improves, which contributes to the enhancement of the removal efficiency at low temperatures. Liang et al. [46] found that when the water–gas ratio is greater than 80, the hydrolysis of COS over the TGH-2 catalyst has a negative effect on the water reaction order. They attributed this to the blocking of micropores due to condensation, halting the reaction process.

(3) Reaction temperature and pressure

Temperature is a crucial factor influencing chemical reaction rates. For COS hydrolysis reactions, an increase in temperature usually contributes to an increase in reaction rate and promotes COS conversion. However, overly high temperatures may lead to catalyst deactivation or an increase in by-product formation.

Pressure may also be a factor in COS hydrolysis reactions under certain circumstances. Although pressure has a relatively minor influence on gas–solid catalytic reactions, changes in pressure may affect adsorption, diffusion, and reaction rate in some specific reaction systems.

(4) Reactant purity

Impurities in the reactants may lead to the deactivation of the catalyst or reduced reaction efficiency, exerting a detrimental impact on the reaction. Consequently, maintaining the high purity of the reactants is one of the crucial factors for ensuring the efficacy of hydrolysis reactions. Huang et al. [43] used γ-Al_2_O_3_ catalyst to simulate industrial stream conditions (CO, CO_2_, H_2_, N_2_, and COS), investigating the effect on COS hydrolysis at 220 °C. Compared with the results of COS hydrolysis without CO/CO_2_/N_2_/H_2_ in the feedstock, the COS conversion rate is slightly lower, at approximately 96%. Wang et al. [47] studied the poisoning effect of oxygen on a hydrolysis catalyst by varying the ratio of O_2_/COS. The more O_2_ is introduced into the reaction system, the higher the probability of collisions between H_2_S and O_2_ on the surface. As a result, the H_2_S conversion rate increases, more S is deposited on the catalyst surface, and the deactivation rate of COS hydrolysis is elevated.

(5) Reaction Time

The duration of the reaction time significantly influences the conversion rate of COS. An overly short reaction time may lead to insufficient conversion, while an excessively long reaction time can increase energy consumption and cost. Hence, the reaction time needs to be optimized to achieve optimal catalytic activity. Thomas [48] reported the effect of Li^+^, Na^+^, K^+^, Cs^+^, Mg^2+^, Ca^2+^, Ba^2+^, and Si^2+^ alkali additives on the hydrolytic activity of COS. The addition of K^+^ increased the hydrolysis activity of the catalyst to over 90%. The initial COS conversion rate of Na+ and Mg^2+^ alkali additives exceeded 95%, but the effect was very short-lived, and the steady-state COS conversion rate dropped to 15–30% after 5 h.

(6) By-product formation and inhibition

During COS hydrolysis, certain by-products, such as CS_2_ and COSO_3_, may be generated. The formation of these by-products will reduce the yield and purity of the target product. Therefore, measures must be taken to inhibit their formation or convert them into harmless substances.

(7) Reactor design and operating conditions

In addition, the reactor design (e.g., flow pattern, temperature distribution, catalyst loading method, etc.) and operating conditions (e.g., gas flow rate, mixing effect, etc.) will also have an influence on the efficacy of the COS hydrolysis reaction. Rational reactor design and optimized operating conditions can improve the reaction efficiency and conversion rate under atmospheric pressure and low temperatures below 90 °C.

## 4. Recent Advances in Catalysts for COS Hydrolysis

Catalysts are typically not composed of a single substance but rather a combination of multiple components. Most catalysts consist of active components, supports, and cocatalysts. In the preparation of catalysts, the selection of catalyst carriers is of paramount significance. A suitable support needs to possess adequate specific surface area, good thermal and mechanical strength, and strong surface activity. Some supports themselves possess certain activity and act as promoters in the reaction.

Additionally, pretreatment can cause the microstructure of the carrier to change to a certain extent. These changes enhance the dispersion of the supported components on the surface of the catalyst carrier, increase the contact between the reaction gas molecules and the active sites, and thereby enhance the catalytic hydrolysis performance of the catalyst. According to the characteristics of catalysts, common COS hydrolysis catalysts can be broadly classified into two categories: supported hydrolysis catalysts and non-supported hydrolysis catalysts. Among these, supported hydrolysis catalysts consist of a carrier with an appropriate specific surface area, good thermal stability, and appropriate mechanical strength, as well as an active component with high hydrolytic activity. Unsupported hydrolysis catalysts are primarily represented by hydrotalcite-like compounds and their derivatives.

### 4.1. Supported Hydrolysis Catalysts

There are two types of supported hydrolysis catalysts that can be used as COS hydrolysis catalyst carriers: those based on metal oxides, such as active γ-Al_2_O_3_, nano-TiO_2_ and ZnO, and those based on non-metallic supports including activated carbon, molecular sieves, and cordierite.

#### 4.1.1. Metal Oxide-Supported COS Hydrolysis Catalysts

Al_2_O_3_, an extensively investigated and widely utilized catalyst, exhibits a large specific surface area, excellent adsorption capacity, high surface activity, and remarkable thermal stability. As a catalyst support material, it not only provides a suitable pore structure for the active component but also enhances the density of alkaline sites essential for COS absorption. Consequently, this improves the catalyst’s activity and selectivity compared to bare alumina in final applications.

The loading of metal oxides onto the catalyst support significantly boosts the number of catalytic hydrolysis active sites on the catalyst surface, transforming the coexistence of acid–base centers into primarily alkaline sites as the dominant active sites. Therefore, loading an appropriate amount of active components onto the support is the main way to improve the catalytic hydrolysis performance of the catalyst. In general, active components are added to the surface of γ-Al_2_O_3_-based catalysts to increase the number and strength of alkaline centers, thereby improving the hydrolysis activity and anti-sulfur and antioxidant capacity of the catalyst [32,35,42,49]. The addition of metals provides different types of active sites that are more suitable for adsorption reactants, promotes catalyst activity, and hinders sulfur deposition or sulfidation on active sites.

Active components refer to substances that interact with reactants, accelerating the rate of chemical reactions towards equilibrium (without altering the equilibrium position) and not being present in the final products. Active components can be single substances or consist of multiple substances. They generally include the support itself, metal oxides, alkali and alkaline earth metals, transition metals, nanometals, and rare earth metal oxides.

A. The carrier itself as the active component

While γ-Al_2_O_3_ typically serves as a support for other metals, which serve as the active sites for general reactions, the hydrolysis of COS is an alkali-catalyzed reaction. The conversion of COS primarily occurs in the presence of alkaline sites, which γ-Al_2_O_3_ can inherently provide. γ-Al_2_O_3_ itself exhibits a certain degree of catalytic activity towards the COS hydrolysis reaction. Shangguan et al. [50] introduced a γ-Al_2_O_3_-based catalyst without any additional active components, achieving a COS hydrolysis conversion rate of approximately 51.2%. Jin et al. [15] synthesized alkali-free ordered mesoporous Al_2_O_3_ with varying pore sizes using a one-pot method for low-temperature catalytic COS hydrolysis. Although sulfur deposition on the catalyst can lead to the loss of catalytic active sites, the m-Al_2_O_3_-3 catalyst with large mesoporous pores inhibited this loss of activity. It can be seen that the expansion of the mesoporous size of the catalyst is crucial to maintaining its catalytic performance. Bachelier et al. [51] investigated the COS hydrolytic activity of three metal oxides in the order of ZrO_2_ > Al_2_O_3_ > TiO_2_, attributing this difference to the abundance and high density of hydroxyl catalytic sites on ZrO_2_ compared to TiO_2_ or Al_2_O_3_. Sulfate impregnation or catalyst sulfonation irreversibly reduces activity, with different deactivation degrees observed at 0.8 µmol·m^−2^ sulfate: Al_2_O_3_ >> TiO_2_ > ZrO_2_. Research by Tang et al. [52] indicated that when γ-Al_2_O_3_ is used as a catalyst carrier under the conditions of a high reaction temperature and moisture content, changes occur in particle size, sintering, microstructure, specific surface area, and pore size and volume, leading to decreased COS hydrolysis activity. Conversely, TiO_2_ demonstrates better heat resistance at high temperatures and greater stability. The hydroxyl content, stability, and chemical activity of Al_2_O_3_ samples prepared by different precursor systems vary. Ning reported [4] that COS was physically adsorbed to saturation on the surface of the catalyst in the initial 20 min, only observing characteristic bands at 2051 and 2071 cm^−1^, indicating that COS was decomposed during catalytic hydrolysis. Increasing the temperature to 90 °C, a new HCO_3_^−^ characteristic peak appeared at 1650 cm^−1^ and gradually disappeared as the reaction time increased, suggesting that COS reacts with the surface hydroxyl group continuously. The hydroxyl content, stability, and activity of Al_2_O_3_-C are the highest, showing the best catalytic performance. The catalytic hydrolysis mechanism of COS was proposed, as shown in Equations (4)–(7).

Al-OH + COS → Al- + HSCO_2_^−^ (4)

2Al-OH + HSCO_2_^−^ → H_2_S + HCO_3_^−^ + Al-O + Al- (5)

Al-OH + HCO_3_^−^ → H_2_O + CO_2_ + Al-O (6)

Al- + Al-O + H_2_O → 2Al-OH (7)

He et al. [53] fabricated three-dimensional ordered macroporous (3DOM) alumina/titania-based COS hydrolysis catalysts with long-range ordered and interconnected hierarchical pores. During the preparation process, the addition of the surfactant P123 increased the surface area of the 3DOM aluminum-based catalyst, while the specific surface area of the 3DOM titanium catalyst was enhanced using a silica stencil method. The porous structure effectively extracts the hydrolysate H_2_S to inhibit the deposition of sulfur on the catalyst surface, thereby enhancing the hydrolysis reaction. Furthermore, in oxygen-containing environments, it was observed that the antioxidant properties of the 3DOM titanium-based catalyst exceeded those of the alumina-based catalyst. Notably, elemental sulfur was found to be predominantly deposited on the surface of the titanium-based catalyst, while sulfate was primarily present on the aluminum-based one. Sulfate not only blocked pore access and damaged alkaline sites on the surface of the catalyst but also reduced the contact between reactants and the catalyst, severely impacting catalytic activity. These findings highlight that employing a 3DOM structure is a promising strategy for improving COS hydrolysis catalysis.

**Figure 3:**
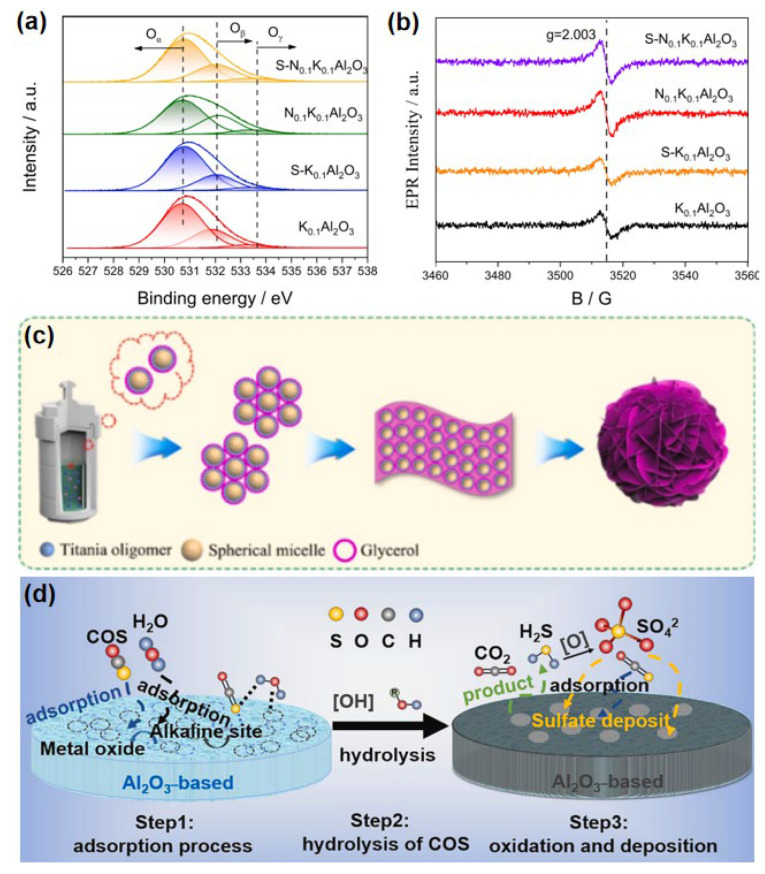
(**a**) XPS spectra of O 1 s and (**b**) EPR results on different catalysts [54]. (**c**) Schematic of the fabrication strategy of a TiO_2_ nanoflower [39]. (**d**) Mechanisms of hydrolysis and poisoning [42]. These figures have been reprinted with permission from the corresponding journal.

The surface basicity plays a crucial role in the catalytic hydrolysis of COS, as hydroxyl oxygen can directly participate in the hydrolysis reaction. This hydroxyl oxygen primarily stems from the OH^-^ groups on the catalyst surface and the dissociation of adsorbed water molecules [12]. As shown in Figure 3a, the nitrogen-doped catalyst exhibits a significantly higher proportion of hydroxyl oxygen (0.067) compared to the unmodified catalyst (0.039), which enhances reaction activity and prolongs catalyst lifespan. EPR experiments (Figure 3b) reveal changes in oxygen species between fresh and poisoned catalysts [54]. After 120 h of the reaction, the N-modified S-N_0.__1_K_0.1_Al_2_O_3_ catalyst shows a higher concentration of oxygen vacancies, which improves the adsorption and activation of reactant gases and ensures long-term stability. DFT theoretical calculations indicate that the main oxidation products of COS are COSO^2-^ and CO_2_SO^-^. The addition of nitrogen makes it more difficult to form oxidation intermediates and facilitates the desorption of SO_2_, suggesting an enhanced resistance to catalyst poisoning [55].

B. Metal/metal oxides as active components

TiO_2_, as the active component, exhibits certain COS hydrolysis activity and demonstrates superior sulfur resistance compared to γ-Al_2_O_3_. However, the hydrolysis process requires relatively high temperatures (100–300 °C), making it challenging to achieve hydrolysis at low temperatures. When TiO_2_ is used as a carrier in a catalyst and subjected to a 400 °C reaction for 1 h, its surface sulfate content is lower than that of γ-Al_2_O_3_. Nevertheless, TiO_2_ has a smaller specific surface area, making it difficult to shape and less suitable for industrial applications. However, a small amount of TiO_2_ modulation significantly enhances the sulfur poisoning resistance of γ-Al_2_O₃ catalysts. Scanning electron microscope observations revealed that the composite carrier was distributed in a granular manner with good dispersion.

Liu et al. [40] leveraged the intrinsic catalytic activity and strong resistance to poisoning of TiO_2_ to prepare an organic sulfur hydrolysis catalyst by loading TiO_2_ onto an Al_2_O_3_ support. The results show that TiO_2_-loaded Al_2_O_3_ exhibited better hydrolysis activity than pure Al_2_O_3_ and demonstrated superior stability due to reduced sulfate formation. The introduction of oxygen vacancies (O*v*) promotes the adsorption and activation of reactants, which is an effective strategy for improving the catalytic performance of COS hydrolysis. Jiang et al. [39] successfully incorporated Cu into the TiO_2_ lattice, inducing more O*v* than pure TiO_2_. As illustrated in Figure 3c, the precursor tetrabutyl titanate undergoes hydrolysis during the evaporation of tetrahydrofuran and subsequently reacts with F127 to form uniform spherical single-molecule vesicles. Subsequently, these TiO_2_/F127 composite vesicles are encapsulated by glycerol. Owing to glycerol’s high viscosity, the predominant collisions between vesicles occur in the parallel direction constrained by the glycerol matrix, leading to the formation of slightly curved two-dimensional nanoplatelets. These nanoplatelets further self-assemble into three-dimensional nanoflower structures. CuTiO_2-δ_-F achieves nearly 100% COS conversion at 70 °C and 93.5% H_2_S yield at 130 °C, outperforming pure TiO_2_. In situ FT-IR tests and density functional theory (DFT) calculations revealed the reaction pathway of COS hydrolysis.

Water activation constitutes the key stage of COS hydrolysis, and the oxygen vacancy defects within the catalyst are capable of regulating water activation. Mu et al. [56] synthesized a series of x-Cu-Co_3_O_4_ nanosheets via the solvothermal method. In situ IR and XPS spectra indicate that the introduction of Cu in Co_3_O_4_ can regulate the oxygen vacancies in the catalyst, thereby expediting the adsorption of COS and the activation of water molecules. The 10-Cu-Co_3_O_4_ sample showed the best activity, and the COS conversion reached 100% at 70 °C. The mechanism of COS hydrolysis involves COS combining with the hydroxyl group to form HSCO^2-^ in the interim and, subsequently, the activated -OH reacting with HSCO^2-^ to generate H_2_S and HCO_3_^2-^. Song et al. [29] carried out experimental and theoretical investigations regarding the simultaneous elimination of COS and CS_2_ over Fe_2_O_3_ and CuO. The synergy of Fe_2_O_3_ + CuO facilitated the removal of both sulfides. Under the circumstances where the COS concentration was 400 ppm and the relative humidity was 49 vol %, with the reaction temperature being 70 °C and the space velocities of Fe_2_O_3_ and CuO being 10,000 and 80,000 h^−1^, respectively, the Fe_2_O_3_ + CuO catalyst achieved a high COS removal rate at a space velocity of 10,000 h^−1^, maintaining a stable COS conversion rate of 100% for the initial 5 h. Experimental data indicated that CS_2_ was initially adsorbed on Fe_2_O_3_, undergoing hydrolysis to form COS, which then migrated from Fe_2_O_3_ to CuO for further COS hydrolysis.

C. Alkali metals and alkaline earth metals as active components

Alkali metals and alkaline earth metals are typically added in the form of metal salts or bases to overcome kinetic restrictions and boost the hydrolytic activity of catalysts. Alkali metals notably adjust the level of alkalinity, while alkaline earth metals govern the distribution of alkalinity strength. Li et al. [57] established a mathematical model for COS removal with catalyst fouling. Kinetic studies were carried out in a fixed-bed reactor at atmospheric pressure and low temperatures (40–70 °C). Based on the experimental results from the breakthrough curve, the kinetic parameters considering axial dispersion, internal and external mass-transfer resistance, and sulfur deposition on inner face of the catalyst can be attained. The model is utilized to fit the breakthrough curve of the COS removal experiment, and the best-fitting parameters are obtained. The adsorption heat of H_2_O and the activation energy of COS removal calculated by the Arrhenius equation are 21.5 and 62.3 kJ/mol, respectively. The model describes the experimental breakthrough curve well and predicts the performance of coupling COS removal. Thomas B et al. [48] impregnated nitrates of different ions (Li^+^, Na^+^, K^+^, Cs^+^, Mg^2+^, Ca^2+^, Ba^2+^, Si^2+^) onto a γ-Al_2_O_3_ carrier via the impregnation method and investigated the effect of alkali doping on COS hydrolysis under low-temperature conditions (20 °C). After 5 h of the reaction, most alkali additives were discovered to be disadvantageous. Only K and Cs at the highest loadings (5 wt %) could slightly enhance COS conversion rates. Meanwhile, the catalysts modified with Na^+^- and Mg^2+^ displayed good initial catalytic activity, with COS removal rates of up to 95%, but this decreased to 15–30% after 5 h. Shangguan et al. [27] prepared the MgAl_2_O_4_ spinel catalyst by dry mixing pseudo-boehmite with magnesium oxide for COS catalytic hydrolysis. The conversion of the MgAl_2_O_4_ catalyst decreased from 99% to 97%, while the COS conversion of γ-Al_2_O_3_ dropped to 87% at the end of the experiment. The authors attributed these results to the larger pore volume and pore size of the MgAl_2_O_4_ spinel catalyst compared to the γ-Al_2_O_3_ catalyst, thus increasing the diffusion rate of COS hydrolysis. The MgAl_2_O_4_ spinel catalyst combines the advantages of γ-Al_2_O_3_ and MgO, showing good catalytic activity for COS hydrolysis and maintaining stability for nearly 30 h at 250 °C and a gas hourly space velocity of 9000 h⁻¹.

Cao et al. [42] extensively screened monometallic and bimetallic formulations on alumina (K/Al_2_O_3_ and K-Me/Al_2_O_3_, where Me = Mo, Zn, Fe, Co, Ni, and Na) and evaluated the oxidation resistance of the most promising samples. Through different potassium precursors doped with alumina, it was found that the catalyst using K_2_CO_3_ performed best. The added quantity of Mo was optimized, and the sample with 5 wt % Mo content was the optimal choice. The authors elucidated the intricate hydrolysis and poisoning mechanism of COS, as illustrated in Figure 3d. Initially, water molecules adsorb onto the active centers of the catalyst, forming alkaline activation sites that facilitate the hydrolysis of COS molecules also anchored at these locations. Notably, COS primarily binds to the hydroxyl groups on the carrier’s surface, initiating a series of transformative reactions. In this dynamic process, the resulting thiocarbonate rapidly decomposes into H_2_S and CO_2_, releasing these gaseous products efficiently. As the reaction progresses, both the oxygen from the feed gas and the lattice oxygen within the catalyst structure engage in oxidation reactions with the adsorbed COS and H_2_S. Over time, sulfur deposition or sulfation progressively deactivates the catalyst’s alkaline centers, reducing its adsorption capacity and alkalinity. This gradual degradation ultimately leads to the complete deactivation of the catalyst, marking the end of its catalytic activity.

The modification of the catalyst with alkaline earth metal has a potential delaying effect on the deactivation of the sulfur poisoning the catalyst. When various active groups are added, the distribution of the alkaline center on the catalyst surface is adjusted, positively impacting catalytic hydrolysis activity. Nimthuphariyha et al. [33] adopted wet impregnation technology to modify a γ-Al_2_O_3_ catalyst with Pt (0.1, 0.5 and 1 wt%) and Ba (2%, 3% and 5 wt%), obtaining a series of Pt/Al_2_O_3_ catalysts doped with barium at an air velocity of 7000 h^−1^ and a reaction temperature of 150−250 °C for COS catalytic hydrolysis. The best performance was attained with 0.5%Pt/5%Ba/Al_2_O_3_, and complete COS conversion was achieved over the entire 10 h test at 200 °C at different COS concentrations. The lifespan of the modified catalyst is 4.5 times that of the original γ-Al_2_O_3_. Xu et al. [58] reported the promotion effect of Mo and K on Al_2_O_3_. In the process of catalyst deoxidation, K can improve the deoxidation capacity of the catalyst, and with the increase in K content, the improvement effect is enhanced. The addition of Mo to alumina reduces the surface alkalinity, which leads to the deterioration of hydrolysis properties. The concentration of H_2_S has no influence on the reduction effect, while the concentration of COS can increase the reduction rate.

D. Transition metals as active components

**Figure 4:**
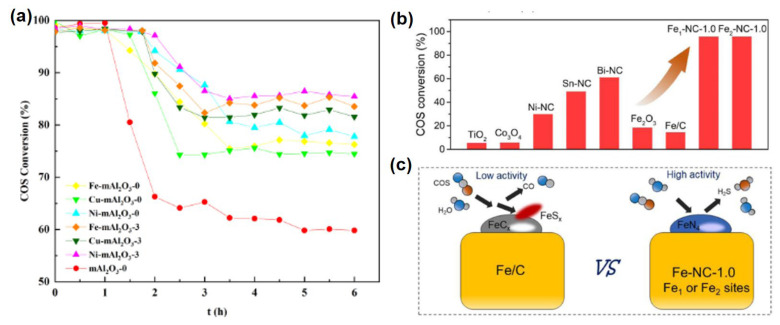
(**a**) COS conversion vs. reaction time for the catalysts incorporated with different transition metals (reaction temperature: 70 °C; COS concentration: 300 ppm; GHSV: 9000 h^−1^; water concentration: 65,553 ppm) [15]. (**b**) Comparison of the COS desulfurization activity of Fe_1_-NC-1.0 and Fe_2_-NC-1.0 with those of various industrial catalysts (test conditions: 400 mg of catalyst, feed gas of 110 mg/m^3^ COS/N_2_, 40 °C of water temperature, 3000 mL/g/h of WHSV). (**c**) Schematic of hydrolysis activity of (**left**) Fe/C and (**right**) Fe-NC-1.0 with isolated Fe sites [59]. These figures have been reprinted with permission from the corresponding journal.

The addition of transition metals can enhance the desulfurization capacity of catalysts by facilitating the combination of metal components with sulfur species on the catalyst surface. The addition of transition metals can transfer the sulfur species adsorbed on the catalyst surface from the weakly alkaline alumina center to the transition metal. Jin et al. [15] synthesized ordered mesoporous Al_2_O_3_-based catalysts for COS hydrolysis, incorporating NiO, CuO, and Fe_x_O_y_ via a one-pot method. When m-Al_2_O_3_ was doped with the same metal—iron, copper, or nickel—the catalytic activity increased with larger pore sizes. By optimizing both pore size and additive type, a highly active and stable large-pore Ni−Al_2_O_3_-3 catalyst was obtained, demonstrating excellent sulfur resistance and maintaining a COS conversion rate exceeding 95% over extended reaction periods (Figure 4a). The minimal deposition of sulfur in the form of elemental sulfur and sulfates on the catalyst surface significantly impacted the activity of small-pore catalysts, while large-pore catalysts retained higher activity levels. Enlarging the pore size and refining the porous structure reduced mass transfer resistance, thereby delaying catalyst deactivation and enhancing catalytic performance. Incorporating iron, copper, and nickel into m-Al_2_O_3_ markedly boosted catalytic activity [38]. These metals, with unfilled outer electron shells, effectively coordinated with sulfur species, facilitating the transfer of deposited sulfur from basic active sites to the doped metals, which further promoted, delayed, or even prevented complete catalyst deactivation.

Tong [44] associated the activity of the Al_2_O_3_ catalyst with the position of the transition metal accelerator M in the periodic table, found the relationship between the catalyst activity and the M-S bonding ability, and concluded that iron as the active component of the catalyst has the highest catalytic activity on COS. Huang [60] used impregnation and co-precipitation methods to load zinc salts onto a γ-Al_2_O_3_ carrier and explored its COS hydrolysis activity via experiments. Initially, the activity of the catalysts with added zinc did not increase significantly. However, as the reaction progressed, the activity of the catalyst rose, indicating that the addition of zinc could inhibit the COS hydrolysis activity of the catalyst in a short period. Introducing oxygen vacancies into metal oxides is an effective strategy for enhancing catalytic activity by facilitating water dissociation and hydroxyl group formation. Zhao et al. [12] doped transition metals (Fe, Co, Ni) onto CeO_2_ to introduce oxygen vacancies and investigated their hydrolysis activity with regard to COS. Their results showed a remarkable improvement in the hydrolytic activity of M/CeO_2_ on COS. The H_2_S selectivity sequence for the four catalysts was found to be Co/CeO_2_ > Ni/CeO_2_ ≥ Fe/CeO_2_ > CeO_2_. DFT calculations revealed that the strong interaction between doped metals (Fe, Co, Ni) and CeO_2_ promotes the spontaneous generation of asymmetric oxygen vacancies in M/CeO_2_. These asymmetric oxygen vacancies facilitate the activation and dissociation of H_2_O as well as the formation of hydroxyl groups, thus exposing more active sites for COS hydrolysis and ultimately increasing its catalytic activity. West et al. [14] carried out research on COS catalysts supported by γ-Al_2_O_3_ and loaded with transition metals (Fe, Co, Ni, Cu, and Zn). Their study demonstrated that all selected metals enhanced the catalytic performance of bare γ-Al_2_O_3_. Nevertheless, this improvement was transient in most formulations; subsequently, the activity of all catalysts declined rapidly except for those doped with Ni and Zn. The Ni and Zn modifiers enhanced the specific activity of γ-Al_2_O_3_ while maintaining its inherent stability. During the initial stages of the experiment, sulfur retention suggested the formation of sulfides on the catalyst surface, which was in line with the findings from deactivated samples. The exception of Ni- and Zn-modified γ-Al_2_O_3_ might be attributed to their stable sulfided surfaces that still allowed for the existence of hydroxyl groups.

E. Rare earth metals/rare earth metal oxides as active components

The addition of rare earth metals can significantly enhance the activity, selectivity, and stability of a catalyst. Specifically, the following advantages are obtained: due to their complex electronic structure with multiple unoccupied electron orbitals, rare earth metals provide a rich amount of electrically active sites, thereby increasing the reaction rate. The electronic structure of rare earth metals can regulate the stability of reaction intermediates, selectively facilitate specific reaction channels, and thereby enhance product selectivity. Rare earth metals have high ion emission capacity and strong oxidation–reduction properties, which enable them to maintain excellent catalytic performance under high-temperature, high-pressure, and other severe conditions. Yang et al. [13] investigated the simultaneous removal of COS and H_2_S at high temperatures using pure SnO_2_ and rare earth metal (Y and La)-doped SnO_2_. A comparison between pure oxides and doped ones showed that the latter had a significantly higher breakthrough sulfur capacity. The larger pore volume and smaller pores of doped SnO_2_, which facilitated better dispersion of Y and La, led to higher COS conversion rates. The sulfur capacity of the 40 wt% LaSnO_2_ sample reached 148.4 mg/g, and the COS conversion rate was nearly 100%. Zhang et al. [61] explored a new rare earth oxide sulfide catalyst for COS hydrolysis. The catalytic activity is closely associated with the difficulty of the rare earth oxides being converted into the corresponding oxidized sulfides. The anti-oxygen poisoning performance of the rare earth oxide sulfide catalyst is evidently superior to that of traditional Al_2_O_3_- and TiO_2_-based catalysts. The competitive adsorption of COS and H_2_O on the surface of rare earth sulfide was discovered. COS and H_2_O engage in competitive adsorption on the catalyst surface, and excessive water will inhibit the chemisorption of COS, leading to a decreased reactivity of adspecies on the catalyst.

γ-Al_2_O_3_ is an effective catalyst and in the absence of CO/CO_2_/H_2_, a considerable amount of elemental sulfur is formed. At 220 °C, the reaction between COS and H_2_O lasts a long time, yet the sulfur balance of H_2_S is approximately 80%. It was discovered that elemental sulfur was deposited on the reactor and catalyst during the reaction. The presence of H_2_ in the reaction increases the sulfur balance to 100%, preventing sulfur deposition on the catalyst. The addition of formic acid to the reactants leads to a reversible loss of activity, but detailed long-term studies have indicated that formic acid is not generated during the operation of the catalyst under a wide range of conditions [43].

#### 4.1.2. Activated Carbon Supported COS Hydrolysis Catalyst

The surface of activated carbon possesses an abundant micropore structure and good electron conductivity. Due to its high specific surface area, large pore volume, various kinds of surface functional groups and high conductivity, it is an ideal carrier for COS hydrolytic catalysts. Currently, the more widely utilized activated carbon mainly consists of two types: commercially available general activated carbon [62,63,64] and biomass activated carbon.

A. General/Modified activated carbon as carriers

In the quest to develop catalysts for water hydrolysis with activated carbon as the carrier, bi-metallic and multi-metallic catalysts featuring double or multiple active components have gradually been engineered to enhance hydrolysis efficiency and durability through the synergistic interactions among various active species. Yi et al. [65] synthesized a bimetallic Fe-Cu modified catalyst and explored the interaction between the two metal oxides. Their results suggested that the optimal water hydrolysis activity was achieved at Fe to Cu ratio of 5:1, as the electron transfer between these metals facilitated an increase in surface active sites. At the same time, this configuration enriched the pore structure of the catalyst, thereby enhancing its hydrolytic performance. He et al. [53] investigated how additives affected the catalytic efficiency of Fe-loaded activated carbon catalysts, disclosing that the optimal conditions were reached when the molar ratio of Fe:Ni was set at 10:1. Ni emerged as a critical factor in regulating the formation of Fe oxide; its incorporation promoted the conversion from metal Fe precursors to metal oxides, which subsequently increased catalytic reactivity. Ning et al. [66] developed a modified coal-based activated carbon with metallic Fe, Cu, and Ce as active components and evaluated its performance in COS catalytic hydrolysis. The outcomes showed that loading with Cu and Ce significantly improved COS hydrolysis activity. Characterization studies indicated that Cu loading affected the microstructural properties, while cerium influenced the formation of Fe oxide, with both positively contributing to the catalytic efficiency. Furthermore, multi-active components regulated product selectivity during hydrolysis by reducing H_2_S oxidation while strengthening the catalyst stability. Notably, integrating La metal elements into the catalyst also had an impact on the overall catalytic performance.

Polymeric carbon nitride (PCN) is a nitrogen–carbon compound with a unique structure and abundant basic sites, whereas metallized PCN (MPCN) is a metal-promoted compound, with its dopants mostly being alkali metals. The study in [67] indicated that MPCN catalysts typically have low initial COS conversion rates, but they are extremely stable over time. As a result, formulations with higher activity, namely K^+^-MPCN and Rb^+^-MPCN, exceeded the K^+^/AC conversion values after 3 h of reaction. Transition elements such as copper, manganese, and zinc were introduced into the catalyst as active components, which influenced the performance of the catalyst in hydrolyzing COS. Yi et al. [68] fabricated a novel metal oxide/activated carbon catalyst by the sol–gel method for COS hydrolysis. The catalyst containing 2.5–5.0 wt.% Fe_2_O_3_ displayed excellent activity after calcination at 500 °C. Among alkaline metal catalysts, the COS conversion rate rose with the increase in the relative density of the alkali load, and its hydrolytic activity followed the sequence of KOH > Na_2_CO_3_ > NaHCO_3_. Qiu et al. [69] developed a Cu-Co-K/activated carbon (AC) adsorbent for COS removal. The modification with CoPcS, Cu(OH)_2_CO_3_, and KOH significantly enhanced the COS removal capability. In TPD tests, the sulfur molecules (H_2_S, COS and SO_2_) were detected in the exhaust gas from the Cu-Co-K/AC adsorbent. It was found that the loss of micropore capacity or the reduction in surface area and volume was negligible, and the spent Cu-Co-K/AC could be effectively regenerated via the thermal desorption process.

Atomically dispersed Fe-N_4_ sites were synthesized by Lei et al. [59] via a straightforward sulfur-mediated approach. The catalytic performance of the optimized iron catalyst was compared with that of other single-atom catalysts, including Co-NC, Ni-NC, Sn-NC, and Bi-NC. Notably, Fe_1_-NC-1.0 and Fe_2_-NC-1.0 exhibited superior activity in COS hydrolysis (Figure 4b). Temperature-programmed desorption studies of CO and H_2_S, along with in situ infrared spectroscopy, indicated that CO and sulfur species easily desorbed from isolated Fe-N_4_ sites. Strong chemical bonding between Fe and C or S increased carbon and sulfur poisoning on the Fe/C surface, thereby inhibiting the COS hydrolysis reaction. The number of iron atoms in the catalyst significantly influenced the adsorption configuration and activation pathway of COS (Figure 4c). Consequently, as the size of iron species increased from single atoms to clusters, the catalytic efficiency decreased due to changes in the mechanism for cleaving the C-S double bond.

B. Biomass-based activated carbon as a carrier

In the 1950s, Dutch scientists discovered the abundance of carbon elements on the Amazonian plains, leading to significant attention being given to biomass carbon. In 2006, the International Biochar Initiative was established, followed by the formation of biochar groups in various regions worldwide. In recent years, biomass-based activated carbon has become a research hotspot in the field of catalytic hydrolysis [2]. Biochar, an adsorbent with a carbon content ranging from 50% to 93%, is obtained through the pyrolysis of biomass. The raw materials for generating biochar can be any natural resources with a high carbon content, such as agricultural residues, woody plants, or animal manure.

**Figure 5:**
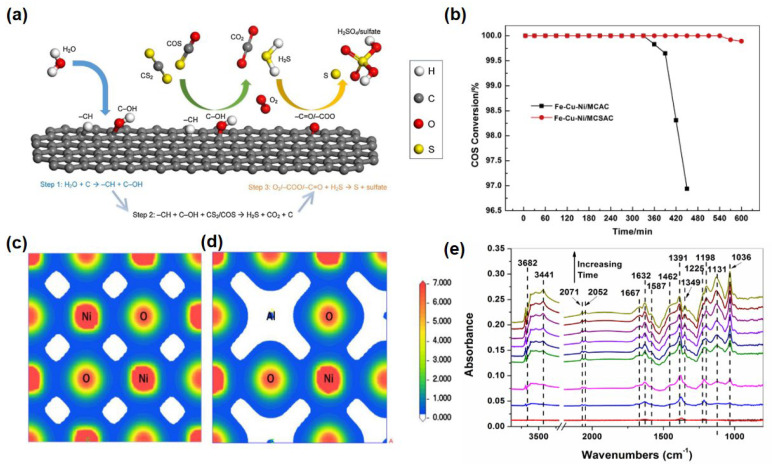
(**a**) Desulfurization process over WSB [70]. (**b**) Simultaneous catalytic hydrolysis of COS over blank MCAC and blank MCSAC catalysts (reaction conditions: 400 ppm COS; 10 ppm CS_2_; GHSV = 10,000 h^−1^; reaction temperature: 50 °C; RH = 49%; O_2_ = 0%) [71]. Electron density of (**c**) NiO and (**d**) Ni_3_Al-HTO [3]. (**e**) In situ DRIFTS spectra of COS removal on NiO [72]. These figures have been reprinted with permission from the corresponding journal.

Song et al. [38] investigated the catalytic performance of walnut shell biochar (WSB) modified with metal oxides and alkaline functional groups for the simultaneous COS and CS_2_. Their study explores the influence of various factors on the calcination effect, such as the type and dosage of metal oxides, the calcination temperature, and the type and content of alkalis. Additionally, kinetic analysis methods were employed to study the mechanism of the hydrolysis reactions. The results demonstrated that Fe_2_O_3_, CuO, and -OH (KOH) were the main active components that catalyzed the hydrolysis of CS_2_ and COS. Song et al. [70] investigated the effect of a biological carbon-based catalyst prepared with walnut shell biochar (WSB) as the raw material on organic sulfur removal. The micropore volume, amorphous structure, and the strength of alkaline sites played crucial roles in the desulfurization process. Strongly alkaline sites were identified as the key factors for chemical adsorption and hydrolysis. Water significantly enhances the desulfurization efficiency on the WSB surface, with hydrolysis being the predominant mechanism under humid conditions. The presence of water results in an increased abundance of CH and C-OH functional groups on the WSB surface, indicating that water molecules can be converted into these groups, thereby promoting the hydrolysis reaction. According to the authors, water first adsorbs onto the WSB surface and subsequently transforms into CH and C-OH groups, which then react with CS_2_ and COS. Additionally, H_2_S is oxidized by oxygen-containing functional groups. The desulfurization process can be summarized as follows and is illustrated in Figure 5a: (1) H_2_O + C → CH + C-OH; (2) CH + C-OH + CS_2_/COS → H_2_S + CO_2_ + C; (3) O_2_/-COO/-C = O + H_2_S → S + sulfate. This sequence elucidates how water facilitates the overall desulfurization reaction pathway. Yi et al. [71] compared microwave coal-based activated carbon (MCAC) and microwave coconut shell activated carbon (MCSAC) supported by Fe-Cu-Ni metal mixed oxides to investigate their catalytic hydrolysis properties. XPS detection showed that most of the products on the waste liquid catalyst were S/SO_2_^4-^, which accumulated on the surface of activated carbon and had adverse effects on hydrolysis activity. To examine the impact of active components on the simultaneous catalytic hydrolysis of carbonyl sulfide (COS) and carbon disulfide (CS_2_), copper nitrate trihydrate (Cu(NO₃)_2_·3H_2_O), nickel nitrate hexahydrate (Ni(NO₃)_2_·6H_2_O), and potassium hydroxide (KOH) were added. The concentration of Fe_2_O₃ was set at 5%, with a molar ratio of Fe:Cu:Ni of 10:2:0.5, and the KOH content was 13%. For the Fe-Cu-Ni/MCAC catalyst, the COS conversion rate reached 100% after approximately 330 min, while for the Fe-Cu-Ni/MCSAC catalyst, it took about 540 min to achieve complete COS conversion (Figure 5b). The sulfur capacities of the Fe-Cu-Ni/MCAC and Fe-Cu-Ni/MCSAC catalysts were measured at 38.54 mgS/g catalyst and 60.52 mgS/g_catalyst_, respectively.

#### 4.1.3. Other Types of Load-Supported COS Hydrolysis Catalysts

Oxysulfides have also been investigated as catalysts for COS hydrolysis because they have higher resistance to the presence of O_2_ and SO_2_ compared with traditional oxides such as alumina or titanium dioxide. Zhang et al. [61] studied several oxysulfide catalysts, especially rare earth metal sulfides for COS. The activity order was La ≈ Pr ≈ Nd ≈ Sm > Eu > Ce > Gd ≈ Ho > Dy > Er. At 200 °C and 5000 h^−1^, the highest-activity catalyst almost completely converted COS. The presence of SO_2_ led to a decrease in COS conversion rate, but the poisoning effect could be mitigated by increasing the reaction temperature. Once the SO_2_ gas was turned off, the catalyst could regain its initial activity. The conversion efficiency decreased slightly when the space velocity was changed from 5000 °C to 20,000 h^−1^.

#### 4.1.4. Non-Supported Hydrolysis Catalysts

In recent years, non-supported hydrolysis catalysts have drawn significant attention, among which hydrotalcite-like compounds (HTLCs) or their modified derivatives are the most widely used as COS hydrolysis catalysts. Generally, HTLCs [73] is a term for anionic clays, also known as layered double hydroxides (LDHs), whose chemical composition is expressed as [M(II)_1−x_[M(III)_x_(OH)_2_]^x+^(An^−^)^x^/n·mH_2_O. M(II) and M(III) denote divalent and trivalent cations, respectively, located within the octahedral structure of the hydroxide layers. The value of x ranges between 0.17 and 0.33, representing the molar ratio of trivalent ions M(III)/M. An- represents the exchangeable interlayer anions [74,75].

A. Hydrotalcite was used as the active component

The metal elements in the hydrotalcite-like structure are adjustable, and thus a variety of hydrotalcite-like catalysts based on metal elements are produced. Wang et al. [1] reported that the catalytic hydrolysis system of COS of mixed oxide catalysts derived from a series of hydrotalcite compounds was studied via the co-precipitation method in a fixed-bed reactor. The M(II)/M(III) ratio is a fundamental parameter affecting the structural characteristics of the HTLC structure, as different cation sizes modify the structure. The influences of pH, synthesis temperature, and hydrothermal treatment on catalytic activity were explored. At 25 °C, the conversion rate of COS was approximately 100%, but it decreased over time for all catalysts. The catalysts effectively removed COS at relatively low temperatures of 50 °C. The results suggest that the pH value and synthesis temperature have an influence on hydrolysis efficiency. A higher synthesis temperature is disadvantageous for the reaction, with an optimal temperature of 25 °C. XRD characterization and catalytic activity indicate that alkalinity is beneficial for COS hydrolysis, where alkaline sites act as active centers to promote the hydrolysis reaction. Li et al. [30] synthesized a series of Cu/Ni/Fe HTO hydrolysis catalysts through a co-precipitation method. The results reveal that the introduction of Cu can enhance the catalytic efficiency, but when the Cu/Ni ratio is 2, the layer structure centered on Cu^2+^ shows significant distortion, resulting in unstable lamellae and a poor crystal phase. Guo et al. [76] synthesized a series of Mg/Al/Ce (HTLcs) via the co-precipitation method with an Al/Ce ratio of 16:1. At a lower temperature of 50 °C, the best removal of COS was achieved with a ratio of 16:1 of Al to Ce. The introduction of Ce increases the hydroxyl group on the catalyst surface, thereby the hydrolysis performance is improved.

The distinctive structural characteristics of hydrotalcite suggest that different preparation methods can markedly influence catalytic hydrolysis efficacy. Mi et al. [77] fabricated Mg/Al layered double hydroxide (LDH) nanosheets by using rapid solid-state mechanochemical processing, characterized by large surface areas and abundant hydroxyl groups, for COS catalytic hydrolysis. These LDH nanosheets exhibited superior activity compared to commercial LDHs and porous metal oxides. Yi et al. [78] investigated how ultrasonic waves influence COS catalytic hydrolysis during the synthesis of a Ni/Al oxide catalyst via co-precipitation from a hydrotalcite framework. The results showed that the ultrasonically prepared hydrotalcites exhibit enhanced particle dispersion and a reduced grain size compared to those produced without ultrasonic assistance in the co-precipitation methods. The pore structure of the hydrotalcite mixed oxide after ultrasonic treatment is more advanced, which is conducive to the physical adsorption of gaseous pollutants. The improved pore structure enhances the diffusion and physical/chemical adsorption of gas pollutants. The effect of ultrasonic treatment on COS removal is highly significant because it promotes the interaction between Ni and Al, which also includes an increase in the number of weakly basic sites (OH^−^) and moderately basic sites (M−O), and a decrease in the number of strongly basic sites (O_2_^−^) responsible for catalyst deactivation. In fact, strongly alkaline sites promote H_2_S oxidation, which leads to the formation of sulfur, resulting in the blockage of the pores of the scaffold.

B. Modified hydrotalc as active component

Given their distinctive structures and properties, composite oxides derived from HTLCs have attracted considerable attention due to their outstanding performance attributes [79]. After high-temperature calcination, hydrotalcites lose crystalline water, along with interlayer anions and hydroxyl groups; this process leads to the disintegration of their layered structures but also leads to an increased surface area after calcination. Furthermore, the catalytic performance of HTLCs depends on pH values during synthesis as well as the calcination temperatures used throughout the production processes. The product generated from COS hydrolysis is H_2_S, which accelerates oxidation reactions involving the presence of H_2_S under oxygen conditions, consequently oxidizing it into elemental S or sulfate forms. Therefore, composite metal oxides originating from HTLCs represent ideal choices for low-temperature COS hydrolysis catalysis. Zhao et al. [31] synthesized a series of Zn-, Ni-, and Fe-like hydrotalcite compounds with varying M^2+^/M^3+^ ratios using the co-precipitation method at pH 10. After calcination at 250 °C, the layered structure of the hydrotalcite was disrupted, generating derived oxides characterized by an increased surface area, reduced mesoporous specific surface area, and enhanced basic sites. However, a further increase in the calcination temperature led to metal oxide agglomeration, resulting in a decreased surface area and pore volume. The oxides derived from hydrotalcite presented three types of basic sites with different strengths: strong basic sites (O_2_^−^ ions), moderate basic sites (M-O pairs), and weak basic sites (OH groups). Characterization techniques such as XRD, FTIR, TG-DTA, SEM, N_2_ adsorption/desorption, and CO_2_-TPD were utilized for analysis. Desulfurization experiments indicated that the Zn-Ni-Fe oxide derivative from hydrotalcite showed remarkable activity in COS hydrolysis at low temperatures.

Zhao et al. [3] examined the hydrolysis performance of a series of Ni-Al hydrotalcite-derived oxides synthesized through diverse preparation methods. The characterization indicated that the urea decomposition method displayed superior catalytic activity in comparison with both the physical mixing method (Ni_3_Al-PM) and the immersion roasting method (Ni_3_Al-IC). The desulfurization activity is closely associated with the surface acid–base properties of the catalyst, and the catalyst prepared by means of the urea decomposition method demonstrated the highest density of basic sites. There is a significant overlap in electron density between O and Ni atoms. The electron density surrounding Ni atoms is substantially higher than that around O atoms, indicating a greater concentration of electrons around Ni atoms [80]. In the case of Ni_3_Al-HTO, there is observable electron density overlap between Ni and O atoms, whereas no such overlap exists between Al and O atoms. Consequently, covalent bonds form between Ni and O atoms, but not between Al and O atoms (Figure 5c,d). This suggests that the intercalated Al atoms are fully ionized.

Generally, COS undergoes initial hydrolysis to H_2_S, which can subsequently be oxidized to elemental sulfur, metal sulfides, or sulfate radicals. The basicity of the derived oxides is determined by their basic sites; the depletion of these sites along with pore blockage are regarded as primary factors contributing to catalyst deactivation. Zhao et al. [81] developed a COS removal catalyst via the calcination of Zn/Ni/Al hydrotalcite and investigated the relationship between structural attributes, acid–base properties, and catalytic performance. Moreover, they noted that both the purity and compositional ratios in zinc–nickel–aluminum hydrotalcite precursors considerably influenced the micropore structure and base site distribution within the catalyst. ZnNiAl oxide exhibited outstanding COS removal efficiency at low temperatures (50 °C), especially when maintaining a Ni:Al ratio of 3:1 for the optimal hydrolytic catalytic activity concerning COS. Density functional theory calculations reveal that there are “numerous defects” in the crystal Ni_3_AlO_4_ (001) plane, which provide more active centers for the chemical adsorption and hydrolysis of COS. Yi et al. [82] calcined hydrotalcite precursors to generate a series of composite oxide catalysts for catalyzing COS hydrolysis reactions. During the calcination process, HTLCs’ structures decomposed into active components such as CoAl_2_O_4_ associated with NiO and NiAl_2_O_4_. The catalyst calcined at 350 °C showed remarkable activity and stability in the COS degradation processes. At this temperature range, the precursors yielded NiO along with spinel phases and hydroxyl groups (-OH), serving as active sites for COS hydrolysis reactions. Conversely, lower roasting temperatures prevent the complete decomposition of precursors into mixed oxides, thereby reducing the overall catalytic efficiency. Meanwhile, increasing the roasting temperature above 350 °C leads to significant sintering among active components like NiO, resulting in the reduction in active site availability; consequently, the formation of elemental sulfur or generation of sulfate contributes further to inactivation. Yi et al. [72] prepared a series of nickel-based hydrotalcite-like compounds through the co-precipitation method. Through the thermal decomposition of the hydrotalcite-like compounds, Ni_3_Al-HTO^-^, Ni_3_Fe-HTO^-^ and Ni_3_Cr-HTO-derived oxides (HTOs) were successfully obtained. It was discovered that the removal activity with regard to carbonyl sulfide (COS) followed the order Ni_3_Al-HTO > Ni_3_Fe-HTO > Ni_3_Cr-HTO. In situ diffuse reflectance infrared spectroscopy and quantum chemical studies revealed the formation of carbonate and sulfate species on the surface of the desulfurization catalysts. The type of trivalent cations had a significant impact on the acid/base properties of HTO, but had no obvious effect on its crystal phase, surface morphology and pore structure. The introduction of trivalent cations reduced the content of Ni^2+^, and the hydrolysis activity of the catalyst was enhanced after the substitution of trivalent cations due to their high electron provision ability. To investigate the precise role of basic sites, in situ DRIFTS experiments were conducted to elucidate the reaction characteristics and analyze the nature of the active centers of the NiO desulfurizer (Figure 5e).

Zhao et al. [75] prepared a novel low-temperature hydrolysis catalyst for COS through the thermal decomposition of Zn-Ni-Al hydrotalcite-derived material. The results indicated that the Zn-Ni-Al hydrotalcite-derived catalyst demonstrated excellent hydrolysis performance at lower temperatures due to the formation of more metal–oxygen bonds (M-O, where M is a metal) in hydrotalcites calcined at 350 °C without altering the number of hydroxyl groups (OH^−^). Both M-O bonds and hydroxyl groups are active sites for COS hydrolysis. The as-prepared sample and HTLCs calcined at a high temperature (500 °C) exhibited low activities. At 500 °C, the pore structures were damaged, and the number of alkaline sites decreased, leading to a reduced COS conversion rate. The calcination temperature had a significant impact on the preparation of hydrotalcite-derived catalysts. The optimal temperature provided more active sites and better pore structures, which favored enhanced catalytic activity. Wang et al. [83] assessed the catalytic performance of cerium-modified Co-Ni-Al hydrotalcites with various Al/Ce ratios of 5, 10, 20, 50, and 60. The CoNiAl-50 catalyst presented optimal activity and stability during COS hydrolysis at 50 °C under the conditions of a space velocity of 2000 h^−1^ and a relative humidity of 2.67 vol%. The incorporation of cerium significantly enhanced the catalytic activity, mainly due to the alterations in structural properties—specifically its unique electronic structure which improves the electron transfer capabilities—as well as the changes in oxidation characteristics and an increase in surface defect sites. Additionally, Ce influenced both the formation of elemental sulfur and the sulfate content, leading to potential catalyst poisoning.

MgAlCe-based hydrotalcites with different Al/Ce ratios, presented by Guo et al. [76] via co-precipitation methods while exploring how operating conditions, including the Al/Ce ratio, pH during preparation, hydrothermal temperature, and calcination temperature, affected the COS removal rates at 50 °C. Lower calcination temperatures maintained the integrity of the layered dual structure; however, calcination above 600 °C undermined the pore structures, resulting in fewer active sites and a decreased COS conversion. The optimal operational parameters for COS removal were identified as pH 8, a hydrothermal temperature of 140 °C, a calcination temperature of 600 °C, and a ratio of 16/1. The catalytic efficiency reached 100%, with a duration of approximately 80 min under low-temperature conditions (50 °C) and a space velocity of 5000 h^−1^. Zhao et al. [79] prepared a series of Zn-Ni-Al-Ce-like hydrotalc compounds via co-precipitation to investigate the effect of their composite oxide on COS hydrolysis. When Al/Ce = 50, the catalytic activity of COS was significantly enhanced; however, excessive cerium doping negatively affected COS hydrolysis by accelerating H_2_S oxidation. XRD, SEM, and EDS results indicated that Ce doping reduced the particle size and increased the dispersion of the catalyst compared to the Ce-free sample. The posited COS hydrolysis catalysts are summarized in Table 1.

## 5. Challenges

**Figure 6:**
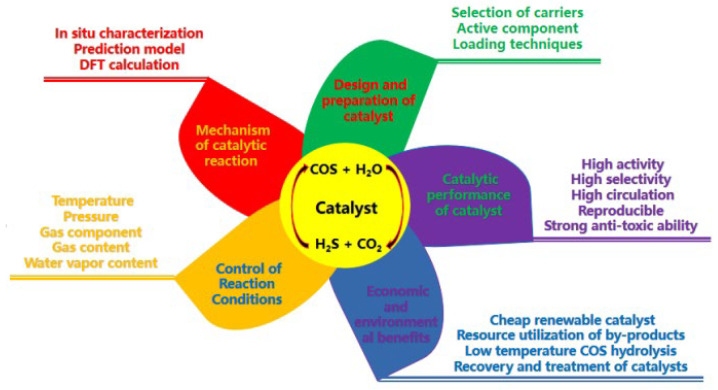
Challenges of photocatalysts COS hydrolysis catalyst.

Although catalytic hydrolysis has demonstrated promising application prospects in the removal of organic sulfur from COS, it still encounters several challenges. Specifically, as illustrated below (Figure 6), these include the following:

(1) Design and preparation of the catalyst: The carriers of COS-hydrolyzing catalysts are mainly classified into metal oxide-based carriers (such as Al_2_O₃ and TiO_2_) and activated carbon-based carriers. The choice of carrier directly influences the activity and stability of the catalyst. Selecting the appropriate carrier and optimizing its structure to enhance the performance of the catalyst is a crucial issue. The loading method and amount of active components like alkali metals, alkaline earth metals, and transition metal oxides have a significant impact on the activity of the catalyst. Therefore, further research is necessary to determine the optimal loading methods and amounts of active components to improve the hydrolysis efficiency of the catalyst.

(2) Control of Reaction Conditions: The hydrolysis reaction requires certain temperature and pressure conditions to balance conversion rates and energy consumption. Optimizing these conditions to enhance conversion rates while minimizing costs presents a challenge. Notably, the water vapor content significantly affects the COS hydrolysis reaction. The conversion rate of COS can be raised by increasing the water vapor content within a certain range, but it will decline after exceeding a certain limit. Precise control of the water vapor content is crucial for optimizing reaction conditions.

(3) Catalytic performance of catalyst: Despite the utilization of various catalysts for COS hydrolysis, the manner of enhancing their activity, selectivity, and stability persists as a pivotal matter. Some catalysts demonstrate high activity under specific circumstances; however, they might be deactivated in practical applications due to fluctuations in reaction conditions. Sulfur species (such as sulfur, sulfite, and sulfate) can be formed on the catalyst surface. The accumulation of sulfur leads to a deterioration of catalytic activity due to the loss of the active sites, while sulfate gradually inactivates the catalyst because of its considerable acidity. Impurities in the raw material gas (for example, O_2_, H_2_S, SO_2_, etc.) can also readily cause catalyst poisoning, reducing its activity and lifespan. Hence, the development of highly resistant catalysts with strong anti-poisoning ability constitutes a crucial research direction.

(4) The mechanism of catalytic reaction: The deactivation of the hydrolysis catalyst results from the reduction in the active surface area caused by sulfur deposition or sulfation, and the deactivation of the catalyst can be mitigated by lowering the reaction temperature. The regeneration method effectively removes sulfur deposits and sulfates from the catalyst surfaces while maintaining their activity and structural stability. Consequently, the development of an effective catalyst regeneration method is of great significance for prolonging the catalyst’s lifespan and reducing production costs.

(5) Economic and environmental benefits: The high production cost of efficient catalysts raises the overall cost of technology application. Simultaneously, the regeneration of catalysts and the disposal of waste catalysts might also impose additional economic burdens. Although the COS hydrolysis reaction can take place at lower temperatures, the overall energy consumption throughout the process still needs to be taken into account. Optimizing the process flow to lower energy consumption is of significant importance. By-products such as H_2_S generated during hydrolysis require further treatment to prevent environmental pollution. The efficient and economic treatment of by-products is among the issues to be tackled in technology applications. Ineffective catalysts must be recovered and disposed of to avoid polluting the environment. Establishing a sound catalyst recovery and treatment mechanism is crucial for ensuring the sustainable development of this technology.

In summary, COS hydrolysis technology confronts certain challenges in the selection and preparation of catalysts, the control of reaction conditions, catalyst deactivation and regeneration, and economic and environmental aspects. To overcome these challenges and promote the widespread adoption of this technology, it is necessary to further intensify basic and applied research, develop low-cost and high-performance catalysts and processes, and establish sound mechanisms for catalyst recovery and disposal.

## 6. Conclusions and Outlook

In recent years, catalytic hydrolysis has witnessed rapid development due to its low energy consumption and minimal side reactions, emerging as the most promising technology for the removal of COS. In this paper, the reaction apparatus (such as a fixed-bed quartz glass reactor using a heating tube furnace), detection technology, influencing factors, evaluation metrics, and reaction mechanisms of COS hydrolysis are comprehensively reviewed. Emphasis is placed on categorizing and introducing the latest research progress of COS hydrolyzing catalysts. Additionally, challenges in this field and potential solutions are discussed. The literature review reveals that COS hydrolysis is a base-catalyzed reaction, where increasing the number of alkaline sites can overcome the kinetic limitations during low-temperature hydrolysis. Furthermore, the reaction is influenced by the porous structure of the employed catalyst. Alumina is the most widely used substance in COS hydrolysis reported so far. It serves as both a carrier and a catalyst. Recent scientific focus has shifted to catalytic materials with special structural properties, including carbon-based materials and mixed oxides, such as layered double hydroxides and hydrotalcite-like compounds. Sulfur species (sulfur, sulfites, and sulfates) can form on the catalyst surface. The accumulation of sulfur leads to a deterioration of catalytic activity due to the loss of the active site, while sulfates gradually deactivate the catalyst due to its pronounced acidity. With stringent requirements for reducing sulfur content in industrial feed gases, new impetus has been injected into the improvement and perfection of existing hydrolysis catalyst preparation methods. Through the study of reaction kinetics and mechanisms, it can provide a better approach for the development of catalysts and the establishment of desulfurization processes. Thus, the focus of reaction kinetics and mechanisms lies in establishing kinetic models under different conditions, determining the influence of various factors, and clarifying the reaction path. In summary, COS hydrolysis is a promising research area that is attracting increasing interest.

Future research directions should focus on the development of novel catalysts, the optimization of reaction conditions, the in-depth study of catalyst deactivation and regeneration, and the exploration of catalytic hydrolysis mechanisms. Specifically, research can be pursued based on the following four aspects:

(1) Exploring new carriers and active components and enhancing the performance and stability of the catalyst through combined optimization. For example, novel materials such as metal–organic frameworks (MOFs) and covalent organic frameworks (COFs) should be investigated as catalyst carriers. Catalysts with anti-poisoning ability should be developed by strengthening their resistance to impurities through doping, coating, and other techniques.
(2) Employing modern computational chemistry methods to simulate the hydrolysis reaction process and optimize the reaction conditions for enhancing the conversion rate and reducing energy consumption. The reaction kinetics and thermodynamic behavior should be studied to disclose the reaction mechanisms and influencing factors, offering theoretical support for process optimization.
(3) Conducting an in-depth study on the mechanisms of catalyst deactivation and regeneration methods and develop efficient and economical regeneration technologies. Enhancing catalyst longevity and developing efficient regeneration strategies are crucial for maintaining consistent hydrolysis efficiency over extended periods. Enhancing catalyst longevity and developing efficient regeneration strategies require a multifaceted approach involving the selection and design of catalysts, the pretreatment of catalyst, the control of operational conditions, and the improvement of regeneration methods. Currently, the regeneration process is assisted by physical means like ultrasound and microwave. Catalyst lifetime prediction models should be explored to provide a scientific basis for catalyst replacement and regeneration.
(4) Combining advanced in situ characterization technology and DFT calculation to clarify the reaction mechanism of the catalyst more clearly at the theoretical level. It is anticipated that the research results of this paper will provide valuable inspiration for researchers engaged in COS hydrolysis.
(5) Translating laboratory-scale advancements in COS hydrolysis catalysts into large-scale industrial applications, requiring careful consideration of reactor design, operational conditions, and economic feasibility. Addressing these challenges will be critical for the successful commercialization of this technology.

## Figures and Tables

**Table 1:** Summary of the reported COS hydrolysis catalysts.

Catalyst	Reaction Condition	COS Hydrolysis Conversion Rate	Magazine Title	Year	Ref.
K_2_CO_3_–Al_2_O_3_–ZnO	Atmospheric pressure; 300 °C; H_2_ 38–42 vol.%, CO 30–33 vol.%, CO_2_ 19–21 vol.%, H_2_O 1–2 vol.%, H_2_S 100–1500 mgS/m^3^, other N_2_ balance.	99.99%	Fuel	2013	[50]
La/SN-Al	200 mg of catalyst (40–60 mesh); 80 °C−280 °C; 150 ppm COS, 100 ppm H_2_S, 0.2% O_2_ and	<100%	*Chem. Eng. J.*	2024	[7]
3D-TS50-K	800 mg/m^3^ COS, N_2_ balanced; RH ^a^: 14.3%.	100% COS conversion lasted for 4 h	*Fuel*	*2019*	[53]
N-doping KAl_2_O_3_	0.5 mL catalysts(40–60 mesh); 0.01 vol% COS, 0.005% CS_2_, 0.5% O_2_ and 5 % H_2_O (balanced by N_2_)	100% COS conversionafter 120 h	*Sep. Purif. Technol.*	2024	[55]
CuTiO_2-δ_-F	0.2 g catalyst (40–60 mesh); 0.1%COS/N_2_; 30–150 °C; under atmospheric pressure (20 mL⋅min^−1^)	100% COS conversion for 10 h at 110 °C	*Chemical Engineering Journal*	2024	[39]
10CueCo_3_O_4_	0.2 g catalyst (40–60 mesh); 30–150 °C; 110 mg m^−3^ with N_2_ as equilibrium gas; WHSV ^b^: 6000–24,000 mL g^−1^ h^−1^;	100% of COS conversion at 70 °C	*Green Energy Environ.*	2023	[56]
Fe_2_O_3_ + CuO	70 °C; 400 ppm COS; 400 ppm CS_2_; relative humidity: 49%; GHSV ^c^:10,000 h^−1^ and 80,000 h^−1^	100% of COS conversion at 400 ppm COS; 49% RH; 70 °C	*Chem. Eng. J.*	2020	[29]
MgAl_2_O_4_	423–523 K; atmospheric pressur COS concentration: 40–220 mg/m^3^; O_2_ concentration: 0.5 vol.%; GHSV: 3000–11,000 h^−1^;	99%	*IOP Conference Series: MSE*	2019	[27]
0.5%Pt/5%Ba/Al_2_O	150–250 °C; COS (1500 ppm in N_2_ balance) was mixed with saturated H_2_O-N_2_	a constant COS conversion at 100% along all the concentrations	*Chem. Eng. Commun.*	2019	[33]
Ni−Al_2_O_3_-3	60−80 °C; 200 ppm COS; H_2_O through a water bath (40 °C, 65,553 ppm); N_2_ balance	95%	*Energ. Fuel.*	2021	[15]
M/CeO_2_	(2.5 vol% H_2_O) and CO (20 vol%)	Not mentioned	*ACS Catal.*	2020	[12]
40% La–SnO_2_	300–400 °C; 0.2% COS and 2.4% H_2_O balanced with N_2_; GHSV: 3000^−1^ h	100%	*Fuel*	2016	[13]
Fe-Cu-Ce/ AC with Fe/Ce = 20	50 °C; COS from gas cylinder (1% COS in N_2_) was diluted with 99.99% N_2_ and 0.5% O_2_ to the concentration of 1500–1750 mg/m^3^; GHSV: 1000^−1^ h	Not mentioned	*J. Rare Earths*	2010	[66]
Cu-Co-K/AC	RH: 0–90%; 20–60 °C; COS: 754; 1129, and 1867 ppm	COS breakthrough adsorption capacity 100.57 mgSg^−1^	*Front. Environ. Sci. Eng.*	2014	[69]
Fe1-NC	50 mg of catalyst; 30 to 90 °C; WHSV: 45,000 mL/g/h;	COS conversion of ca. 95.9% at 90 °C	*ACS Catal.*	2024	[59]
walnut-shell biochar catalysts	15 ppm CS_2_ and 500 ppm COS; GHSV: 10000 h^−1^; 5 °C water with 49% RH	100%	*Appl. Sur. Sci.*	2017	[70]
Fe–Cu–Ni/MCAC	50 °C; 400 ppm COS; 10 ppm CS_2_; O_2_ = 0% GHSV: 10,000 h^−1^; RH: 49%	100% COS conversion for about 330 min	*Chem. Eng. J.*	2013	[71]
precipitating solution of (Co + Ni)/Al at pH 9.0	50 °C, GHSV = 3000 h^−1^, temperature of saturator: 25 °C	COS conversion 99% about 100 min at 25 °C	*Chem. Eng. J.*	2011	[1]
Mg_2_Al	50 °C; 470 ppm COS; N_2_ to balance; RH: 2.67%; GHSV: 5000 h^−1^;	98% conversion for 60 min	*RSC Advances*	2015	[76]
30−70 °C	30−70 °C; WHSV: 6000–48,000 mL/(g·h);	reaches 100% at 70 °C	*Chem. Commun.*	2019	[77]
NiAl-HTO	25 °C; 200 ppm COS; The total gas flow rate was 20 mL/min, with a catalyst volume of 0.2 mL	100% after 30 min	*Ultrason. Sonochem.*	2016	[78]
Zn-Ni-Al-Ce	50 °C; 1600 mg/m^3^ COS; 0.5% O_2_; N_2_ to balance; GHSV: 5000 h^–1^; RH: 3.0%	98%	*J. Rare Earths*	2010	[79]
Zn/Ni/Al HTO	50 °C; 350 ppm COS; 250 ppm–300 ppm CO_2_; N_2_ to balance; GHSV: 3000 h^−1^;	100%	*Catal. Today*	2019	[81]
CoNiAl Catalysts (T350)	50 °C; 950–1000 mg·m^−3^ COS; N_2_ to balance; GHSV: 2000 h^−1^; RH: 2.67%	95% conversion could sustain for more than 700 min	*Ind. Eng. Chem. Res.*	2011	[82]
Ni_3_Al-HTO	60 °C; 200 ppm COS	100%	*Mater. Chem. Physics*	2018	[72]
Zn–Ni–Al HTLCs calcined at 500 °C	50 °C; 360 ppm COS; 0.5% O_2_ in N_2_; N_2_ to balance. GHSV: 5000 h^−1^;	95%	*Catal. Commun.*	2011	[75]
CoNiAl-50	50 °C; 1016 mg·m^−3^ COS, N_2_ to balance. GHSV: 2000 h^−1^; RH: 2.67%	99% conversion for 210 min	*Appl. Clay Sci.*	2012	[83]
potassium promoted NiAl hydrotalcite-derived oxides	100 mg catalyst; 60 °C; 670 mg/m_3_ COS; the total gas flow was 25 mL/min	Not mentioned	*Sep. Purif. Technol.*	2018	[28]
Ni_3_Al-HTO	60 °C; COS from gas cylinder (1% COS in N_2_) was diluted with 99.99% N_2_ to the concentration of 1000 mg/m^3^; GHSV: 5000 h^−1^	100%	*Catal. Today*	2020	[32]
N_0.1_K_0.1_Al_2_O_3_	0.3 g catalyst (40–60 mesh); 0.02 vol% CS_2_, 0.01 vol% COS and 0.5 vol% O_2_; 5 vol% H_2_O	48.23% COS conversion after 16 h	*Fuel*	2024	[41]

^a^ RH: relative humidity; ^b^ WHSV: weight hourly space velocity; ^c^ GHSV: gas hourly space velocity.

## Data Availability

The original contributions presented in this study are included in this article; further inquiries can be directed to the corresponding author.
